# DiGeorge Syndrome Gene *tbx1* Functions through *wnt11r* to Regulate Heart Looping and Differentiation

**DOI:** 10.1371/journal.pone.0058145

**Published:** 2013-03-22

**Authors:** Priya Choudhry, Nikolaus S. Trede

**Affiliations:** 1 Huntsman Cancer Institute, Department of Oncological Sciences, University of Utah, Salt Lake City, Utah, United States of America; 2 Department of Pediatrics, University of Utah, Salt Lake City, Utah, United States of America; Institute of Cellular and Organismic Biology, Taiwan

## Abstract

DiGeorge syndrome (DGS) is the most common microdeletion syndrome, and is characterized by congenital cardiac, craniofacial and immune system abnormalities. The cardiac defects in DGS patients include conotruncal and ventricular septal defects. Although the etiology of DGS is critically regulated by *TBX1* gene, the molecular pathways underpinning *TBX1*'s role in heart development are not fully understood. In this study, we characterized heart defects and downstream signaling in the zebrafish *tbx1^−/−^* mutant, which has craniofacial and immune defects similar to DGS patients. We show that *tbx1^−/−^* mutants have defective heart looping, morphology and function. Defective heart looping is accompanied by failure of cardiomyocytes to differentiate normally and failure to change shape from isotropic to anisotropic morphology in the outer curvatures of the heart. This is the first demonstration of *tbx1*'s role in regulating heart looping, cardiomyocyte shape and differentiation, and may explain how Tbx1 regulates conotruncal development in humans. Next we elucidated *tbx1*'s molecular signaling pathway guided by the cardiac phenotype of *tbx1^−/−^* mutants. We show for the first time that *wnt11r* (wnt11 related), a member of the non-canonical Wnt pathway, and its downstream effector gene *alcama* (activated leukocyte cell adhesion molecule a) regulate heart looping and differentiation similarly to *tbx1*. Expression of both *wnt11r* and *alcama* are downregulated in *tbx1^−/−^* mutants. In addition, both *wnt11r* ^−/−^ mutants and *alcama* morphants have heart looping and differentiation defects similar to *tbx1^−/−^* mutants. Strikingly, heart looping and differentiation in *tbx1^−/−^* mutants can be partially rescued by ectopic expression of *wnt11r* or *alcama*, supporting a model whereby heart looping and differentiation are regulated by *tbx1* in a linear pathway through *wnt11r* and *alcama*. This is the first study linking *tbx1* and non-canonical Wnt signaling and extends our understanding of DGS and heart development.

## Introduction

DiGeorge syndrome (DGS) is the most common microdeletion syndrome occurring in 1/4000 live births [1]. Approximately 75–80% of patients have congenital heart disease with conotruncal defects (Tetralogy of Fallot, aortic arch defects, and truncus arteriosus) and ventricular septal defects. Other defects include thymic hypoplasia, palate defects and thyroid and parathyroid abnormalities. Cardiac defects are the leading cause of mortality in DGS, but the underlying molecular pathobiology is not well understood.

Most DGS patients have a 3 Mb or a nested 1.5 Mb deletion of chromosome 22q11.2 that includes the *TBX1* gene. Genetically engineered mouse mutants have led to the identification of *Tbx1* as the gene responsible for cardiovascular and thymic defects [2], [3], [4]. Several patients without the chromosomal deletion harbor frame-shift and missense mutations in *TBX1*, strongly suggesting that DGS is caused by haploinsufficiency of *TBX1* [5], [6]. *TBX1* is a member of a group of transcription factors that are characterized by the presence of a T-box, a highly conserved DNA-binding region that also has a conserved interaction domain for other transcription factors [7]. T-box genes mediate transcriptional activation and/or repression and are important for embryonic development and differentiation of all three germ layers [8], [9], [10]. In addition, T-box genes are extremely dose-sensitive and often act in a combinatorial or hierarchical fashion [7].

Dose-sensitivity of mouse *Tbx1* gene has been tested using various hypomorphic and null alleles and transgenic models [3], [11], [12]. While *Tbx1+/−* mice have a milder phenotype, *Tbx1−/−* mice have a severe phenotype with single cardiac outflow tract (OFT), aortic and pharyngeal arch defects, thymus and parathyroid gland aplasia [2]. Tissue-specific knockdown of *Tbx1* in the pharyngeal endoderm or mesoderm recapitulates the cardiac defects observed in *Tbx1−/−* mice and DGS patients, making it difficult to study the role of *Tbx1* separately in different tissues [13], [14]. Previous studies have identified several genes, including *Fgf10*, as part of the *Tbx1* pathway [12]. However, validation and detailed description of their roles downstream of *Tbx1* during development is lacking.

While non-canonical *Wnt* signaling has no known link to *Tbx1*, it inhibits β-catenin signaling and promotes its own signaling through protein kinase C and c-jun terminal kinase during normal heart morphogenesis [15], [16], [17], [18]. *Wnt11*, a non-canonical *Wnt* member, is required for heart specification and can induce expression of cardiac genes in *Xenopus* explants and non-cardiac cells from humans and mouse [15], [19], [20], [21], [22], [23], [24]. Furthermore, mutations in *Wnt11* cause cardiac OFT defects such as truncus arteriosus, similar to those observed in *Tbx1−/−* mutants [25]. Knockdown of *wnt11r*, an ortholog of *Wnt11* present in *Xenopus*, resulted in heart morphology defects and cardia bifida in some cases [15]. In spite of the similarity in phenotypes caused by loss of *Tbx1* and *Wnt11* signaling, no link between these pathways has been established to date.

The pattern of heart development is conserved through evolution. Development begins with specification of the bilateral Primary Heart Fields (PHF) in lateral plate mesoderm. Subsequent migration towards the midline results in the cardiac crescent and later the linear heart tube, at which point the heart starts beating. The tube then undergoes asymmetric looping and morphogenetic movements to result in the multi-chambered heart. This complex process involves ballooning of the chamber walls, addition of cardiomyocytes derived from the Secondary Heart Field (SHF) at the arterial pole and formation of septa and valves. Formation of heart chambers is accompanied by differential gene expression within the heart [18], [26]. Cell tracing experiments in mice and chick have established that heart myocardium derives from PHF, while SHF contributes to the OFT [27]. Interestingly, previous studies suggest that *Tbx1* is expressed in SHF and that *Tbx1* positively regulates SHF cell proliferation and contribution to the muscle layer of OFT [12], [28], [29].

This vertebrate pattern of heart development is largely conserved in zebrafish. Similar to mice and chick, zebrafish PHF cells migrate towards the midline to form a linear heart tube [30]. Moreover, presence of SHF and its contribution to OFT has been recently confirmed in zebrafish [31], [32]. Zebrafish have a simple two-chambered heart with a single inflow and outflow tract and looping results in an S-shaped heart instead of the 4-chambered heart with multiple outlets in higher vertebrates [30]. The OFT retains the bulbous arteriosus (BA), an accessory chamber equivalent to the conus arteriosus of amphibians and reptiles. This structure has been replaced by the pulmonary and aortic trunk in mammals and birds [33], [34]. The simpler structure of the heart combined with transparency of embryos and availability of a *tbx1* mutant, make zebrafish the ideal system to study cardiac defects associated with DGS. Here we use the zebrafish *tbx1^−/−^* mutant [35] to study cardiac defects associated with DGS and identify *tbx1* target genes. We show that *tbx1* is required for normal heart looping and differentiation of myocardium derived from the PHF and SHF. In addition we provide evidence that *tbx1* mediates its function at least in part via *wnt11r*, and one of its downstream mediators, *alcama*.

## Materials and Methods

### Fish stocks and maintenance

Fish were maintained at 28.5°C under standard conditions [36] and were staged as described [37]. The *vgo^tm208^* (*tbx1* ^−/−^) line and *wnt11r ^fh224/+^* (*wnt11r^−/−^*) mutant lines were a kind gift from Dr Tatjana Piotrowski (Stowers Institute of Medical Research, Kansas City, MO) and Dr. Cecilia Moens (Fred Hutchinson Cancer Center, Seattle, WA). Homozygous mutants were obtained by inbreeding of heterozygous carriers. *Tg(cmlc2:EGFP)* fish that express green fluorescent protein (GFP) in the nuclei of cardiomyocytes under the control of *cardiac myocyte light chain* promoter, were a gift from Dr Joseph Yost (Department of Neurobiology, University of Utah) [38].

### Identification and genotyping of mutants

*tbx1^−/−^* mutants have an A-to-T missense mutation [35]. Mutant embryos were identified by high resolution melting analysis [39]. DNA was extracted from the tails of stained embryos and PCR was conducted using the primers 5′- CACAACTGAAAATCGCCAGCAATC-3′ (forward, 0.2 µM), 5′- AATATGGTAAAACTCACCAGTCCT -3′ (reverse, 1 µM) and 5′- TTTACCAAAGGCTTCAGAGACTGTAATCCC -3′ (unlabelled probe complementary to reverse strand, 1 µM). After PCR, the samples were heated to 94°C for 2 minutes and then cooled to 10°C before melting. LCGreen dye (Idaho Technology) was included in the PCR mix and melting analysis was done on LightScanner™ (Idaho Technology). The *wnt11r^−/−^* mutant was generated by tilling, and has a G-to-T nonsense mutation that generates a stop codon at amino acid 94. *wnt11r^−/−^* mutants were identified in a similar fashion using the primers 5′- GGTCTGCCAAAAGACCTTCACAG-3′ (forward, 0.2 µM), 5′- TTGGAAATAAATGCGTTTAGACACGGTT-3′ (reverse, 1 µM) and 5′- TGTTCCCCTATTGATGGACCGAAACTCCT-3′ (unlabeled probe complementary to reverse strand, 1 µM). All the identified WT and mutants were included in the analysis.

### Cloning and RNA transcription

The *tbx1* gene cloned in pCS2+, and *wnt11r* gene cloned in pCMVSport6.1, were obtained from ZIRC (Zebrafish International Resource Center). To make sense RNA for injection, these plasmids were cut with *NotI* and *XhoI* respectively, and in vitro transcribed from Sp6 promoter using mMessage Machine kit (Ambion). RNA for *alcama* was made as previously described [40].

### Morpholino antisense oligonucleotide and RNA injections

Morpholino and RNA were dissolved in molecular biology grade water and pressure injected into 1–4 cell zebrafish embryos. For RNA rescue experiments, 26 pg of *tbx1*, *wnt11r* or *alcama* RNA was injected per embryo. The previously described translation blocking morpholino for *alcama* was used at 1.1 ng per embryo [40].

### Tissue labeling procedures

Whole mount RNA in situ hybridization (ISH) with digoxigenin was performed as previously described [41]. The plasmids for *versican* and *eln2* were a gift from Dr Joseph Yost (Department of Neurobiology, University of Utah) and for *amhc* and *vmhc* from Dr Dean Li (Department of Human Genetics, University of Utah). Alcama protein was stained using Zn-5 antibody from ZIRC at 1∶500 dilution. A goat anti-mouse secondary antibody conjugated with Alexa 555 (Invitrogen) was used for fluorescence labeling of cell boundaries. For non-fluorescence detection, secondary antibody conjugated to alkaline phosphatase (Bio-Rad) was used with NBT-BCIP (Roche). The Isl-1 antibody was a gift from Dr Richard Dorsky (Department of Neurobiology and Anatomy, University of Utah) and was used at 1∶100 dilution. Labeling of bulbous arteriosus with DAF-2DA (VWR) was done as previously described [42].

### Imaging and quantification

Head-on photographs of 48 hours post fertilization (hpf) larvae were taken on Nikon SMZ1000. Dissected hearts and ISH embryos were photographed on a Nikon Y-IDP microscope at 20× zoom using Spot software. Confocal images of dissected hearts were taken on Olympus FV1000 microscope at 40× zoom. Images of all larvae from the same experiment were taken using the same settings and exposure. Cell numbers were counted using Imaris software. Measurements for circularity, length and volume were done using ImageJ and angles were measured on Adobe Photoshop. Live embryos were mounted in 1% low melt agarose and movies of the beating heart were taken with the Nikon Y-IDP microscope.

### Morphometric measurements

Head-on pictures of *cmlc2* stained 48 hpf embryos were used for measuring looping angles. To quantitate the looping angle, we measured the angle between the longitudinal axes of ventricle and atrium as shown in schematic Figure S1D. The ventricular axis was drawn by connecting the midpoint at the dorsal end of the ventricle (m, where it meets the OFT) and the midpoint at the widest part of the ventricle in a parallel plane to the dorsal end (n). Similarly the atrial axis was determined by joining the midpoint at the ventral end of the atrium (p, where it meets the IFT) and the midpoint at the widest part of the atrium in a parallel plane to the ventral end (q). The length of the chambers was determined by measuring along the longitudinal axes (m-n-o for the ventricle, and p-q-r for the atrium) with the end point at the bisection of the arc formed by the boundary of *cmlc2* staining near the AVC (o and r, respectively).

The BA is shaped like a circle sliced horizontally at dorsal and ventral ends. Width of the BA was measured at the widest part of the small circular chamber (diameter of the circle). The length was measured from the ventral end of the cut where it meets the ventricle, to the dorsal end where it continues as an artery.

### Analysis of cardiac performance

Movies of beating hearts were imaged as above and analyzed as follows. Heart rate was calculated by counting the number of sequential contractions. The widths of ventricles/atria at diastole and systole were measured. The width was measured at the widest part along the anterior-posterior axis. Since the chambers are prolate spheroids, width measurement along the anterior-posterior axes in lateral view is equivalent to left-right measurement in a head-on view. Shortening fractions were calculated as width [(diastole – systole)/diastole]. Stroke volume was determined as previously described [43]. The perimeter of the ventricle during diastole and systole was outlined on ImageJ and analyzed with a “fit-to-ellipse” algorithm, giving the major and minor axes. Volume was calculated using V = 4/3 * π * a * b^2^, where “a” is the major axis and “b” the minor axis. The stroke volume was obtained by subtracting the volume at systole from volume at diastole.

### Paraffin embedding and sectioning

72 hpf larvae were fixed in 4% paraformaldehyde for 2 days, dehydrated in ethanol series and transferred directly to xylene. They were allowed to equilibrate for 90 min and then placed in paraffin. Embryos were embedded in disposable plastic molds and cooled before sectioning at 5 µm on a Leica RM2155 microtome. Glass slides were heated to 60°C overnight, placed in xylenes for 5 min, rehydrated through an ethanol series to water, stained with 0.1% toluidine blue, and coverslipped using Cytoseal 60 (Fisher Scientific).

### Ethics statement

This work has been approved by the University of Utah IACUC (#11-07006). All the fish are housed at the campus fish facility where, all laboratory personnel are trained for the maintenance and care of zebrafish. I am trained and certified for handling zebrafish.

## Results

### *tbx1* is required for normal cardiac looping in zebrafish

**Figure 1:**
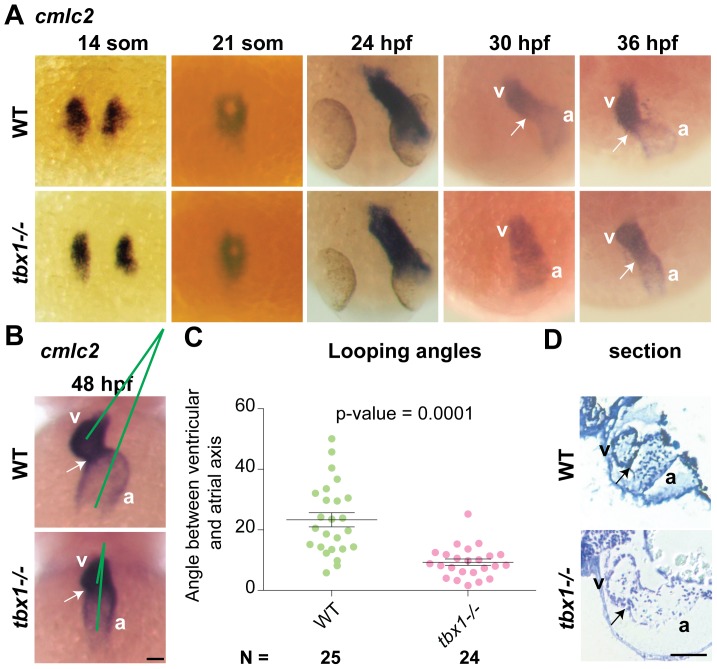
*tbx1* expression and heart looping defects in *tbx1^−/−^* embryos. (A) *cmlc2* ISH in *tbx1^−/−^* mutants and WT siblings at specification (14 somites), fusion (21 somites), linear heart tube (24 hpf), jogging (30–36 hpf) stages. (B) By 48 hpf the heart has finished looping in WT forming an acute angle between the atrial and ventricular axes (green lines), while in *tbx1^−/−^* mutants the axes are nearly parallel. (C) Comparison of atrio-ventricular axis angles between wild-type (left, green) and *tbx1^−/−^* mutants (right, red). N indicates the total number of embryos represented in the plot. (D) 5 micron sections of the hearts from 72 hpf embryos, showing wider ventricle and atrium and thinner heart walls in *tbx1^−/−^* mutant as compared to WT sibling. Arrows indicate the AVC; a, atrium; v, ventricle. Scale bar: 50 µm.

In zebrafish, *tbx1* expression initiates between 12–14 hpf in the lateral plate mesoderm containing the cardiac progenitors [35], and is maintained in the myocardium as the heart develops (Fig. S1A–C). By 48 hpf, expression is stronger in the ventricle as compared to the atrium and particularly pronounced in the atrioventricular canal (AVC) (Fig. S1C). We investigated whether *tbx1* is necessary for correct cardiac morphogenesis in zebrafish, similar to humans and mice. To that end, we visualized cardiomyocytes in the *tbx1^−/−^* null mutant [35] using *cmlc2 in situ* hybridization (ISH). The cardiac progenitors are correctly specified at 14 somites, migrate towards the midline to fuse at 21 somites and form the linear heart tube at 24 hpf in *tbx1^−/−^* mutants. However, jogging of ventricle (30–36 hpf) and looping of the heart (36–48 hpf), is defective in *tbx1^−/−^* mutants resulting in a straight heart (Fig. 1B) rather than the WT S-shaped heart (Fig. 1A). The straight heart phenotype was variable between larvae, so we measured the angle between the longitudinal axes of ventricle and atrium to quantify the defect (see Experimental Procedures and Fig. S1D). There is a statistically significant decrease in the angle between WT (22°) and *tbx1^−/−^* mutants (10°) (Fig. 1C). While the atrioventricular angle and left-right positioning of the heart is affected in *tbx1^−/−^* mutants, the antero-posterior positioning of the heart is unaffected. Our data demonstrate that *tbx1* is necessary for heart looping.

### *tbx1* regulates cardiomyocyte shape

**Figure 2:**
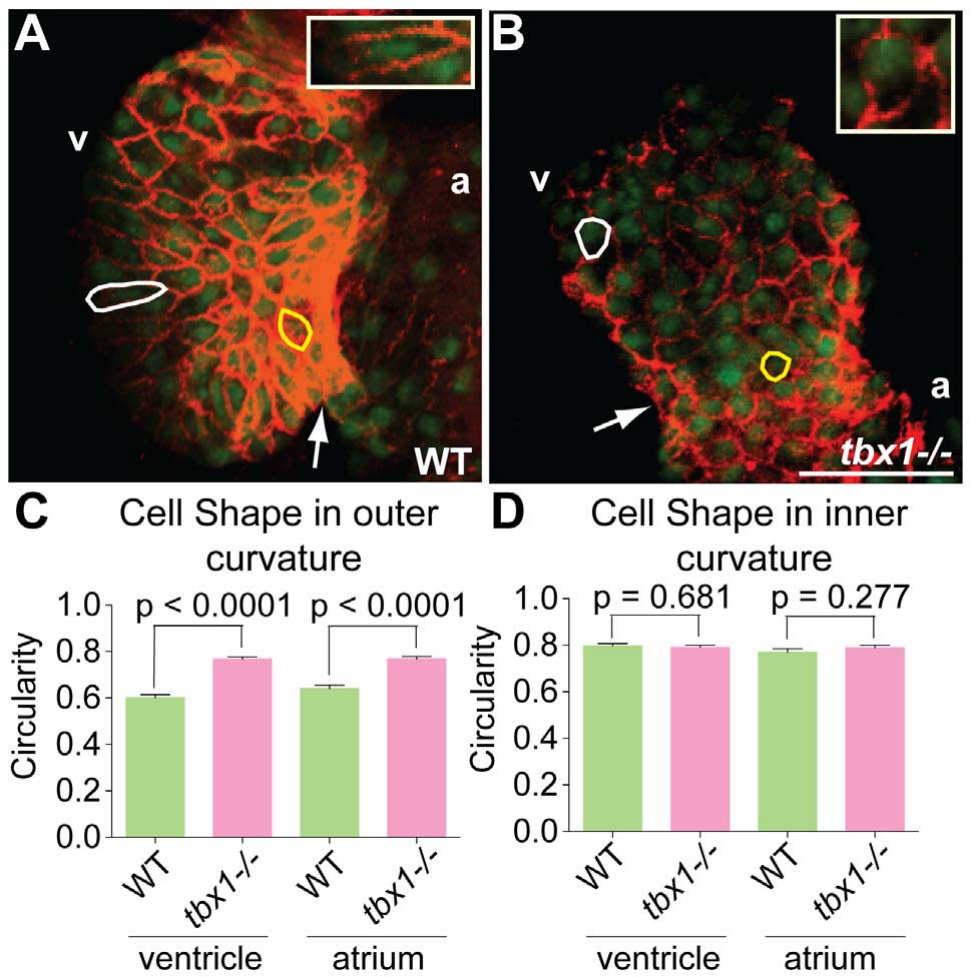
Cell shape defects in *tbx1^−/−^* mutants. (A, B) Confocal projections of hearts from *Tg(cmlc2:EGFP)* embryos at 48 hpf stained with Alcama antibody (red) to demarcate cell boundaries. Cells in the outer and inner chambers are outlined in white and yellow respectively. The insets in E and F show a magnified view of the outer curvature cell outlined in white. The plot in C shows that cells in the outer chamber of *tbx1^−/−^* mutants are rounder (circularity tending towards 1) as compared to WT. (D) Cell shape is unchanged in the inner chamber of *tbx1^−/−^* mutants. Each bar in plots C and D represents data collected from 70 cells in 7 different embryos. Arrows point to the AVC; v, ventricle; a, atrium. Scale bars: 25 µm.

Cardiomyocyte shape changes are a major contributor to normal heart looping [44], [45]. During the process of looping, the cells transition from small and rounded (isotropic) morphology in the linear heart tube (24–28 hpf) to flattened and elongated anisotropic cell shapes in the outer curvature at the expanded chamber stage (48–58 hpf) [46]. We investigated the possibility that *tbx1* regulates the shape of cardiomyocytes, hence affecting heart looping. To that end, we stained 48 hpf *Tg(cmlc2:EGFP)* larvae with Alcama antibody to visualize the cell boundary, and analyze cell shape. Larvae were co-stained with DAPI to visualize the nuclei, and hearts were dissected and imaged using a confocal microscope (Fig. 2A, B). In WT siblings, the cells in the outer curvature are elongated with their longitudinal axes pointing towards the AVC (white outline), while cells in the inner curvature continue to be small and rounded (yellow outline) (Fig. 2A). However, *tbx1^−/−^* mutant cells in the outer and inner curvatures retain the small and rounded morphology at 48 hpf (Fig. 2B). We quantified this defect by calculating the circularity of 10 cells from the inner and outer chambers of 7 mutant and WT embryos. Cells from *tbx1^−/−^* mutants in the outer curvature have 1.2–1.3 fold higher circularity (are rounder) as compared to WT siblings (Fig. 2C), while cells in the inner curvature are equally round between the mutant and WT (Fig. 2D). In summary, our data suggest that *tbx1* is required for regulation of cardiomyocyte shape, and heart looping defect in *tbx1^−/−^* mutants.

### *tbx1* regulates cardiac morphology and cardiomyocyte number

In addition to the looping defect, *tbx1^−/−^* mutants have other cardiac morphological defects. Mutant ventricles are significantly shorter at 48 hpf (Fig. S1E), while atrial length is similar between mutants and WT. The ventricle at its widest point is significantly wider in the mutant when compared to WT at 48 hpf, (Fig. S1F) and 72 hpf (data not shown). Atrium width differences between mutants and WT are significant at 72 hpf (Fig. S1G), but not at 48 hpf (data not shown). In addition, sections of the heart at 72 hpf, reveal that mutant hearts have thinner ventricular walls and larger intercellular spaces (Fig. 1D).

The smaller ventricle size in *tbx1^−/−^* mutants may be due either to fewer or to smaller cardiomyocytes. To distinguish between these possibilities, we stained *tbx1^−/−^; Tg*(*cmcl2:EGFP*) larvae with DAPI to count total cell numbers in hearts (Fig. S2A,B). Total cardiomyocyte number was unchanged at 33 hpf, but was significantly decreased 1.3 fold at 48 hpf (post-looping and morphological defects) (Fig. S2C). This correlates with the lower cell density and larger intercellular spaces observed in sections of *tbx1^−/−^* mutant hearts (Fig. 1D). Our data suggests that *tbx1^−/−^* mutants have proper specification and differentiation of cardiomyocytes during early stages marked by *cmlc2* expression (Fig. 1A), but have decreased number of cardiomyocytes during later stages (after 33 hpf, Fig. S2C).

The late decrease in cardiomyocyte number observed in *tbx1^−/−^* mutants may be caused by decrease in proliferation of the PHF cells. We tested this possibility by analyzing proliferation of cardiomyocytes using phosphohistone 3 (PH3) staining to determine the percentage of cells undergoing mitosis (Fig. S2A, B). Analysis of PH3 staining reveals that the mean number of proliferating cells per heart is significantly reduced 1.6 times at 33 hpf (15 in WT siblings versus 9 in *tbx1^−/−^* mutants, Fig. S2D). However, the low percentage of cardiomyocytes undergoing mitosis (5–8%) combined with the physiologic decrease in cell proliferation from 24 to 48 hpf [47], suggests that decreased proliferation in *tbx1^−/−^* mutants might be insufficient to drive the dramatic difference in cardiomyocyte number at 48 hpf (235 in WT siblings versus 194 in *tbx1^−/−^* mutants).

An alternative or additional explanation of reduced cardiomyocyte number in *tbx1^−/−^* mutants is reduced contribution of SHF-derived cells. Recently several groups have reported the existence of a SHF in zebrafish that contributes cardiomyocytes to the arterial pole of the heart tube between 24 and 48 hpf [31], [32], [48]. During the course of our analysis, Hami et al. reported that *tbx1^−/−^* mutants have reduced incorporation of dye-labeled cells from the pericardial wall to the arterial pole of the heart. Thus the reduced cardiomyocyte number in *tbx1^−/−^* mutants could result from a combination of decrease in PHF cell proliferation and decrease in incorporation of SHF cells. Taken together, these data suggest that *tbx1* regulates proliferation of PHF cells and incorporation of SHF cells into the developing heart. Whether SHF cell incorporation plays a role in heart looping is an intriguing possibility and will be a part of future investigations.

### *tbx1* expression is necessary for regional differentiation of heart

At 48 hpf the heart becomes differentiated to form the various regions: inflow tract (IFT), atrium, atrioventricular canal (AVC), ventricle and OFT. In zebrafish, heart looping defects are highly correlated with defects in regional differentiation of the heart, suggesting that these regional changes in expression pattern are important for the looping process [46], [49], [50], [51], [52], [53]. We performed ISH analysis in *tbx1^−/−^* mutants at 52 hpf to evaluate if regional differentiation occurs normally. The ventricular marker *vmhc* and atrial marker *amhc* are expressed normally in *tbx1^−/−^* mutants, suggesting that atrial and ventricular specification occurs normally in *tbx1^−/−^* mutants (Fig. S3).

**Figure 3:**
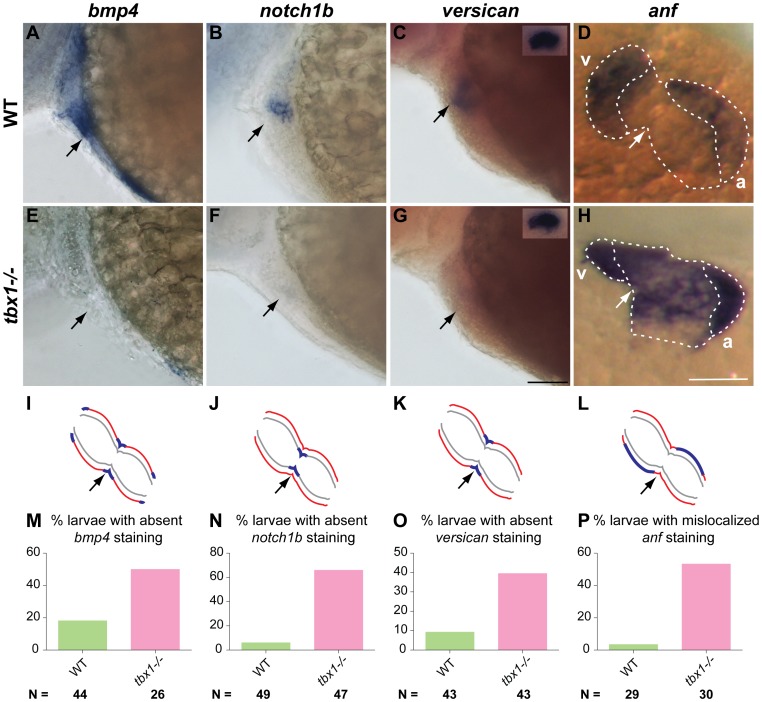
Regional differentiation defects in *tbx1^−/−^* mutants. 48 hpf embryos stained for region-specific markers (A–H) and their schematic representation (I–L). Red lines indicate myocardium, grey lines indicate endocardium and blue lines indicate gene expression. *bmp4* (OFT, AVC and IFT), *notch1b* (AVC endocardium) and *versican* (AVC myocardium) expression is down-regulated in *tbx1^−/−^* mutants (E–G) as compared to WT siblings (A–C). The insets in C and G show *versican* expression in the ear as a control for ISH. *anf* expression is localized to the outer parts of the chamber myocardium in WT (D), but is broadly expressed in the ventricle and atrium in *tbx1^−/−^* mutants (H). Arrows point to the AVC in all panels; v, ventricle; a, atrium. The plots in M–P show the penetrance of the phenotype as percentage embryos with absent/mis-localized expression of the respective gene. N depicts the total number of embryos represented in the plot. Scale bars: 50 µm.

We next analyzed expression of markers specific to different regions of the myocardium. At 52 hpf, *bone morphogenetic protein 4* (*bmp4*) is expressed in the IFT, AVC and OFT in WT siblings (Fig. 3A), but expression is absent in *tbx1^−/−^* mutants (Fig. 3E). Similarly, WT siblings express *versican* and *notch1b* in the AVC myocardium and endocardium, respectively (Fig. 3B, C). However, *tbx1^−/−^* mutants fail to express these markers in the AVC at this stage (Fig. 3F, G). Next, we analyzed *atrial natriuretic factor* (*anf*), whose expression is restricted to the outer curvature of the ventricle and atrium in WT siblings (Fig. 3D). However, in *tbx1^−/−^* mutants, *anf* expression is expanded to the inner curvature in the ventricle and to a lesser degree in the atrium. In order to determine if these defects are heart specific, we examined *versican* expression in the otoliths. *tbx1^−/−^* mutant otoliths express *versican* (inset Fig. 3G), indicating that *tbx1* is required for regional expression of these markers specifically in the heart.

The abovementioned markers are expressed broadly along the linear heart tube at 30 hpf, but become restricted to specific regions by 48 hpf. These markers are expressed normally in the *tbx1^−/−^* mutants at 30 hpf (data not shown), indicating that the differentiation defect arises later in development, concomitant with the heart looping and cardiomyocyte shape defects. The differentiation defect may be an effect or a cause of the looping defect. In addition, penetrance of these defects is not uniform in *tbx1^−/−^* mutant larvae (Fig. 3M–P). Alterations in *notch1b* expression showed the highest penetrance and we will henceforth use it as a marker for regional differentiation. Failure of *tbx1^−/−^* mutants to restrict *anf* expression, taken together with absence of *bmp4*, *notch1b*, and *anf*, suggests that *tbx1* is necessary for regional differentiation of the heart.

### *tbx1* is necessary for normal OFT formation and differentiation

**Figure 4:**
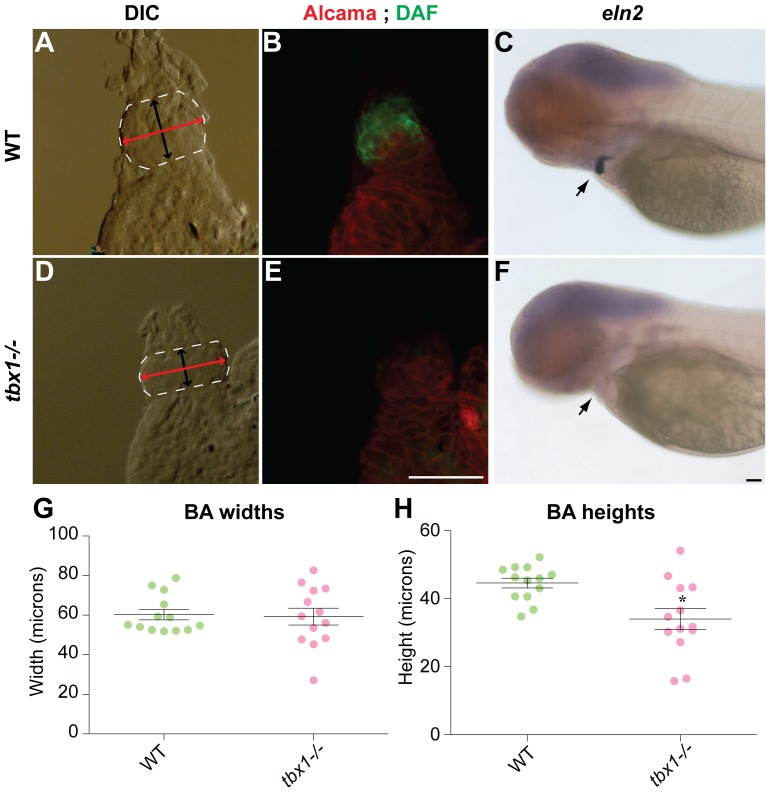
Bulbous arteriosus defects in *tbx1^−/−^* embryos. Dissected hearts from 72 hpf embryos showing the BA region (A, D) outlined in white, with the bidirectional arrowheads in black and red showing the length and width of BA, respectively. (B, E) The corresponding images showing staining for Alcama antibody (red) and DAF-2DA (green). (C, F) *tbx1^−/−^* mutants at the same stage have absent *eln2* expression. Black arrows indicate the BA. (G) Quantification of BA width in WT and *tbx1−/−* mutants, while (H) demonstrates that BA length is reduced in *tbx1^−/−^* mutants; * p-value = 0.0045. N = 13 for all measurements. Scale bars: 25 µm.

DGS patients and *Tbx1−/−* mutant mice have defects in OFT such as persistent truncus arteriosus and interrupted aortic arch. Hence we studied the OFT in the zebrafish *tbx1^−/−^* mutants. The zebrafish ventricle ends in a constriction leading to the BA, an accessory chamber present in lower vertebrates that is composed of smooth muscle. The BA is shaped like a circle sliced horizontally at the dorsal and ventral ends, where it meets the artery and ventricle, respectively (Fig. 4A, D; for details of measurement parameters, please refer to the “morphometric measurements” in the Materials and Methods section). While BA width is unaffected (Fig. 4 G), its length is reduced in *tbx1^−/−^* mutants versus WT siblings (Fig. 4H).

In addition to morphology, we analyzed the *tbx1^−/−^* mutant for proper differentiation of the BA. To that end we used the fluorescent nitric oxide sensor DAF-2DA, a marker for smooth muscle [42]. Our analysis revealed that BA smooth muscle in *tbx1^−/−^* mutants is defective as evidenced by negative staining with DAF-2DA (Fig. 4B, E). *tbx1^−/−^* mutants also fail to express the BA-specific marker *tropoelastin2* (*eln2*) at 72 hpf (Fig. 4F). Taken together, our findings suggest that in the absence of *tbx1* signal, the BA is underdeveloped and undifferentiated. In keeping with our data, a recent report demonstrated that *tbx1^−/−^* mutants have reduced incorporation of SHF cells at the arterial pole [32], which may contribute to decreased size of BA.

### *tbx1^−/−^* mutants have defects in cardiac performance

To determine how the heart looping and differentiation defects in *tbx1^−/−^* mutants affected cardiac function, we analyzed movies of beating hearts in live embryos at 31, 48 and 72 hpf. Cardiac contractility was assessed using the ventricular and atrial shortening fractions. Mean ventricular shortening fraction was unaffected at 48 hpf but was significantly lower at 72 hpf in *tbx1^−/−^* mutants (0.160±0.012) compared to WT (0.197±0.014) (Fig. S4A). In contrast, mean atrial shortening fraction was unaffected at 48 hpf and 72 hpf (Fig. S4B). As shortening fraction is a function of width, the unchanged atrial contractility was in keeping with the weaker morphological defect observed in *tbx1^−/−^* mutant atria (Fig. S1E, G). Considering the decreased ventricular contractility in *tbx1^−/−^* mutants, we next assessed the cardiac output as stroke volume of the ventricle. While the stroke volume was 10–20% lower in *tbx1^−/−^* mutants at both 48 and 72 hpf, this difference is not statistically significant (p-value >0.05) (Fig. S4C). The unchanged stroke volume suggests that in spite of decrease in ventricular length, the mutant heart probably compensates by increased ventricular width to maintain cardiac output.

In addition to contractility and stroke volume, another important factor to measure for cardiac performance is the heart rate. Heart rate was lower in *tbx1^−/−^* mutants at both 48 and 72 hpf. At 48 hpf the mean heart rate decreased to 75% in *tbx1^−/−^* mutants (108±2 beats per minute) as compared to WT siblings (143±2 beats per minute). At 72 hpf the mutant heart rate (167±2) partially recovered to 86% of WT (195±4) (Fig. S4D). This is in direct contrast to DGS patients where the heart beats faster to compensate for OFT obstructions. Since zebrafish do not have any obstruction in the BA, they probably do not need this compensatory mechanism. In conclusion, the ventricle in *tbx1^−/−^* zebrafish mutants has defects in contractility and heart rate, while stroke volume is unaffected.

### *wnt11r* regulates heart looping and regional differentiation similar to *tbx1*

While several genes have been implicated in the *tbx1* pathway, detailed characterization of the *tbx1* pathway regulating DGS phenotype is lacking. We sought to utilize the zebrafish heart looping defect to identify new genes that may function downstream of *tbx1* during this process. To that end we utilized a candidate gene approach, and analyzed all genes known to regulate heart looping and whose misregulation cause cardiac diseases in humans. Interestingly, T-Box proteins frequently interact and several among them, such as *tbx2a*, *tbx3b* [50], *tbx5* [53], and *tbx20* [54], have been implicated in zebrafish heart looping. Furthermore, mutations in *TBX5* and *TBX20* are associated with congenital heart disease in humans. To test whether these genes function downstream of *tbx1* to regulate zebrafish heart development, we analyzed the expression of these *tbx* genes in the *tbx1^−/−^* mutant. By ISH analysis, all these genes are more highly expressed in the ventricle as compared to the atrium in WT embryos at 52 hpf. However, these genes are unaffected in the *tbx1^−/−^* mutants (Fig. S5A–D, F–I), suggesting that they are not regulated by *tbx1*.

**Figure 5:**
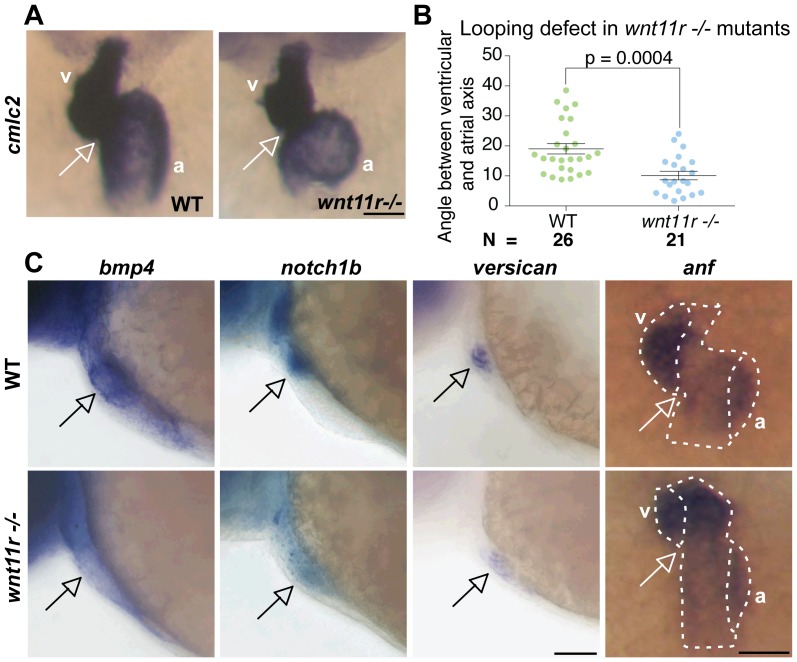
Heart looping and regional differentiation defects in *wnt11r^−/−^* mutants. (A) *cmlc2* ISH showing the looping defect in *wnt11r^−/−^* mutants. (B) Atrio-ventricular axis angle in wild-type (left, green) and *wnt11r^−/−^* mutants (right, blue). N indicates the total number of embryos from 3 experiments. (C) Defective regional differentiation of heart in *wnt11r^−/−^* mutants, similar to those seen in *tbx1^−/−^* mutants. Scale bars: 50 µm.

Another important gene that regulates heart development and looping is *Wnt11*. In spite of similarity in cardiac phenotype of *Tbx1−/−* and *Wnt11−/−* mouse mutants, no link between these genes has been established to date. While zebrafish *wnt11r* is homologous to *Xenopus wnt11r*, and *Xenopus wnt11r* has been implicated in heart looping [15], the role of zebrafish *wnt11r* in heart development and looping has not been studied to date. We sought to test whether *wnt11* may function in the *tbx1* pathway using zebrafish mutants. Hence we first studied heart development in the zebrafish *wnt11r^−/−^* mutant [55]. *cmcl2* ISH revealed that the *wnt11r^−/−^* mutants also have straight hearts, similar to *tbx1−/−* mutants (Fig. 5A). Quantification of angle between the ventricular and atrial axes revealed that the looping angle is significantly reduced to 10° in *wnt11r^−/−^* mutants from 19° in WT siblings.

Next, we analyzed the regional differentiation in *wnt11r−/−* hearts. Similar to *tbx1−/−* mutants (Fig. 3), *wnt11r^−/−^* mutants have unchanged ventricular and atrial differentiation (data not shown). However, similar to *tbx1−/−* mutants (Fig. 3), *wnt11r^−/−^* mutants have down-regulated *bmp4*, *versican* and *notch1b* (Fig. 5C). Additionally, expression of *anf* is expanded to the inner curvature especially in the ventricle (Fig. 5C). Staining with DAF-2DA and *eln2* ISH reveal that *wnt11r^−/−^* mutants do not have BA defects (Fig. S6), suggesting that the heart looping and regional differentiation defects are independent of BA defects. Together, the heart looping and regional differentiation defects observed in *wnt11r* ^−/−^ mutants indicate that *wnt11r* may function downstream of *tbx1* during these processes.

### *tbx1* and *wnt11r* function in the same pathway to regulate heart looping and regional differentiation

**Figure 6:**
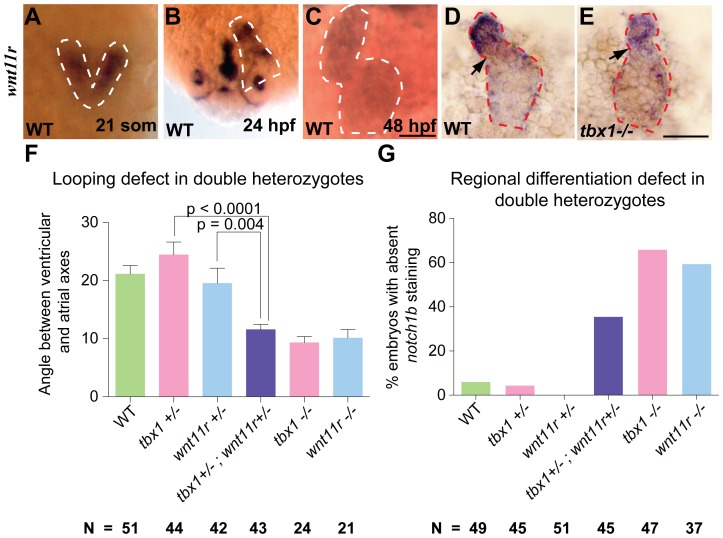
*wnt11r* and *tbx1* cooperate to regulate heart looping and differentiation. (A–C) ISH showing *wnt11r* expression in the fusing heart fields (21 somites, A), linear heart tube (24 hpf, B) and fully looped heart (48 hpf, C) in WT embryos. (D, E) Dissected hearts from WT (D) versus *tbx1^−/−^* mutant (E) embryos stained for *wnt11r* expression at 48 hpf. The heart boundaries are indicated by dotted lines. Arrows point to the AVC. Scale bars: 50 µm. (F, G) Quantification of penetrance of looping and regional differentiation defects in double heterozygotes as compared to single heterozygotes of *tbx1* and *wnt11r*. WT is shown as negative control, and *tbx1^−/−^* and *wnt11r^−/−^* mutants as positive control for looping and regional differentiation defects. N depicts the total number of embryos from 3 experiments.

The expression of *wnt11r* in zebrafish heart development in zebrafish is unknown. Our ISH analysis revealed that *wnt11r* is first expressed in cardiac mesoderm just prior to fusion (21 somites, Fig. 6A) and after *tbx1* expression in the heart begins. *wnt11r* expression is maintained in cardiomyocytes in both the atrium and the ventricle as the heart forms and loops (Fig. 6B). Over time, *wnt11r* expression weakens throughout the heart, although it is still present as late as 48 hpf (Fig. 6C). The expression of *wnt11r* in the heart correlates with the straight heart defect in *wnt11r* ^−/−^ mutants. Furthermore, the initiation of *wnt11r* expression after the onset of *tbx1* expression in cardiac progenitors supports the possibility that that *tbx1* initiates heart expression of *wnt11r*.

**Figure 7:**
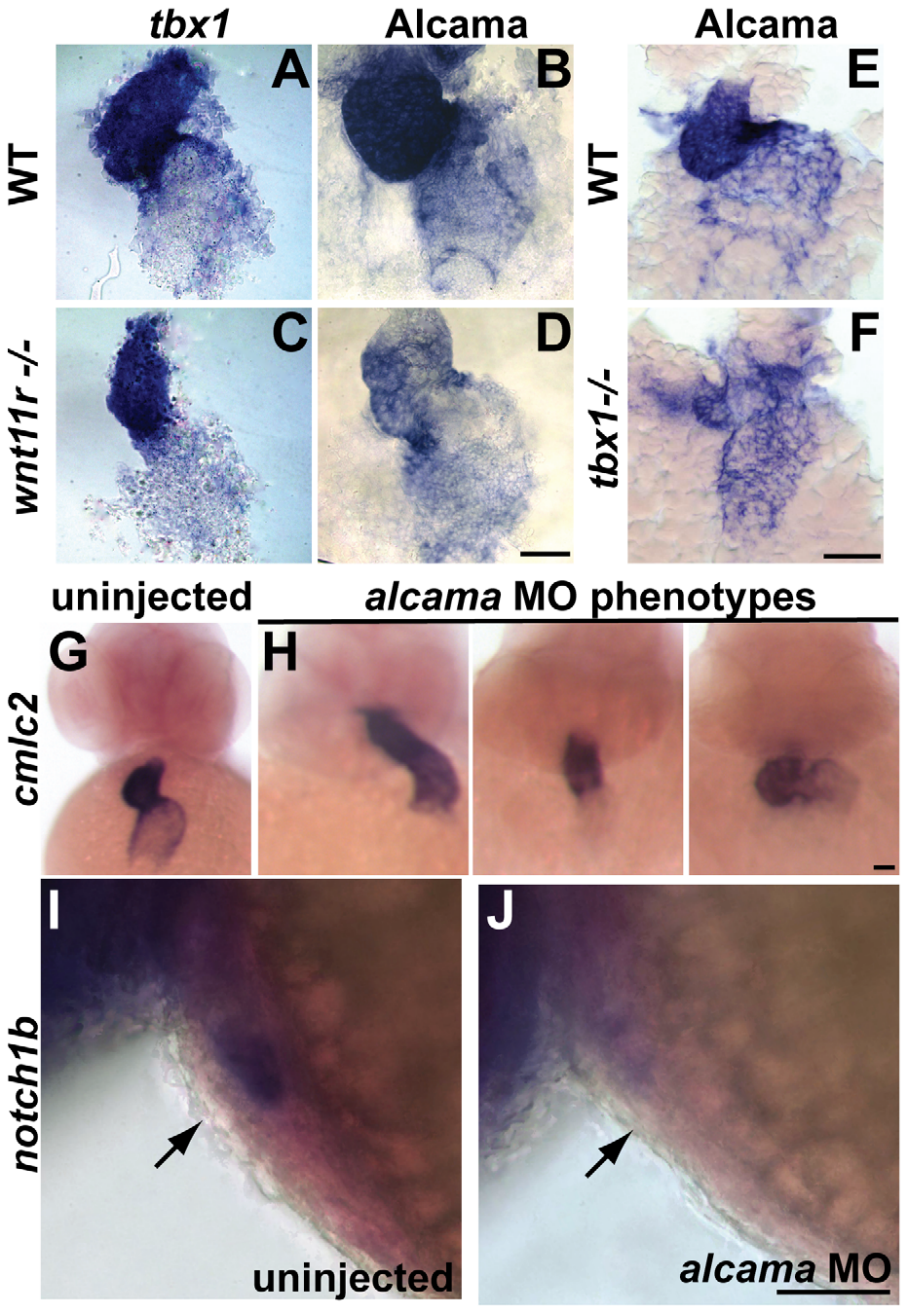
*tbx1* and *wnt11r* regulate Alcama levels, and *alcama* regulates heart looping and differentiation. (A, C) ISH analysis on 48 hpf embryos showing that *wnt11r^−/−^* mutants have unaffected *tbx1* expression. (B, D, E, F) Antibody staining showing that *wnt11r^−/−^* (D) and *tbx1^−/−^* (F) mutants have down-regulated Alcama expression when compared to WT siblings (B, E). *cmlc2* ISH on 48 hpf embryos showing the various looping phenotypes observed in *alcama* morphants (H) and proper looping in an uninjected larva (G). *notch1b* is expressed in the AVC endocardium in an uninjected larva at 48 hpf (I), but is down-regulated in *alcama* morphants (J). Scale bars: 50 µm.

To assess whether *tbx1* regulates *wnt11r* during heart development we analyzed *wnt11r* expression in *tbx1* knockdowns. If *tbx1* activates *wnt11r* expression, we would expect a decrease in *wnt11r* signal in the absence of *tbx1*. Indeed, we observed that *wnt11r* expression is down-regulated in *tbx1^−/−^* mutants (Fig. S5E, J and 6D, E). This result was corroborated by quantitative RT-PCR, which indicates that *wnt11r* RNA is down-regulated by 37% in hearts of *tbx1^−/−^* mutants (not shown). In contrast, *tbx1* expression is unchanged in *wnt11r^−/−^* mutants as compared to WT siblings (Fig. 7A, C). The defective heart looping and regional differentiation defects of *wnt11r* ^−/−^ mutants, the timing and location of *wnt11r* expression with respect to *tbx1* expression, and the reduction of *wnt11r* in *tbx1^−/−^* mutant larvae all support our hypothesis that *tbx1* regulates heart looping and regional differentiation through *wnt11r*.

To further explore this hypothesis we conducted non-allelic non-complementation assays. *tbx1^+/−^* and *wnt11r^+/−^* heterozygotes have unaffected cardiac looping and regional differentiation (Fig. 6F, G). We crossed *tbx1^+/−^* and *wnt11r^+/−^* mutants and analyzed the double heterozygous larvae. Our analysis reveals that *tbx1^+/−^;wnt11r^+/−^* larvae have incomplete looping and fail to express *notch1b* in significantly more larvae as compared to *tbx1^+/−^* or *wnt11r^+/−^* larvae (Fig. 6F, G). The penetrance of defects in *tbx1^+/−^;wnt11r^+/−^* is slightly lower than *tbx1^−/^* ^−^ or *wnt11r^−/−^* mutants, but the resulting phenotype is the same. Thus the double heterozygote carrying recessive mutations in *tbx1* and *wnt11r* exhibits a phenotype similar to homozygous mutant of either gene, while single heterozygotes do not. These double-heterozygote analyses indicate a case of non-allelic non-complementation and suggest that *tbx1* and *wnt11r* function in the same pathway to regulate heart looping and regional differentiation.

### *tbx1* regulates heart looping and regional differentiation via *wnt11r* and *alcama* in a linear pathway

Previous work identified *Xenopus alcama* as a downstream target of *wnt11r* during differentiation of cardiomyocytes and heart looping [16]. This led us to propose *tbx1* regulates heart looping and differentiation by activating *wnt11r*, which in turn activates *alcama*. We tested our hypothesis by analyzing Alcama expression during heart development. *alcama* expression starts in the heart progenitors at 21 somites and continues on until 4 dpf [56], [57]. Similar to *tbx1* expression, Alcama protein expression is stronger in the ventricle than atrium and is prominent in the AVC in WT embryos (Fig. 7B, E). However in both *tbx1^−/−^* and *wnt11r^−/−^* mutants, Alcama expression is strongly down-regulated in ventricles and weakly down-regulated in atria at 48 hpf (Fig. 7D, F), supporting our hypothesis that *alcama* is downstream of *tbx1* and *wnt11r*.

To test the specific function of *alcama* during zebrafish heart morphogenesis, we injected a morpholino directed against *alcama* [40] into zebrafish at the 1-cell stage and assessed heart looping and regional differentiation at 48 hpf. *alcama* morphants have defective heart looping of varying severity (Fig. 7H), a finding that may be explained by dosage differences in morpholino injections. In addition to looping defects, *alcama* morphants have defects in regional differentiation of the heart as assessed by *notch1b* ISH (Fig. 7J). Furthermore, in accordance with our hypothesis that *alcama* functions downstream of *tbx1* and *wnt11r*, their expression is unaffected in *alcama* morphants (data not shown). Hence, we have shown that Alcama is down-regulated in *tbx1^−/−^* and *wnt11r^−/−^* mutants, and that *alcama* is needed for normal heart looping and differentiation.

**Figure 8:**
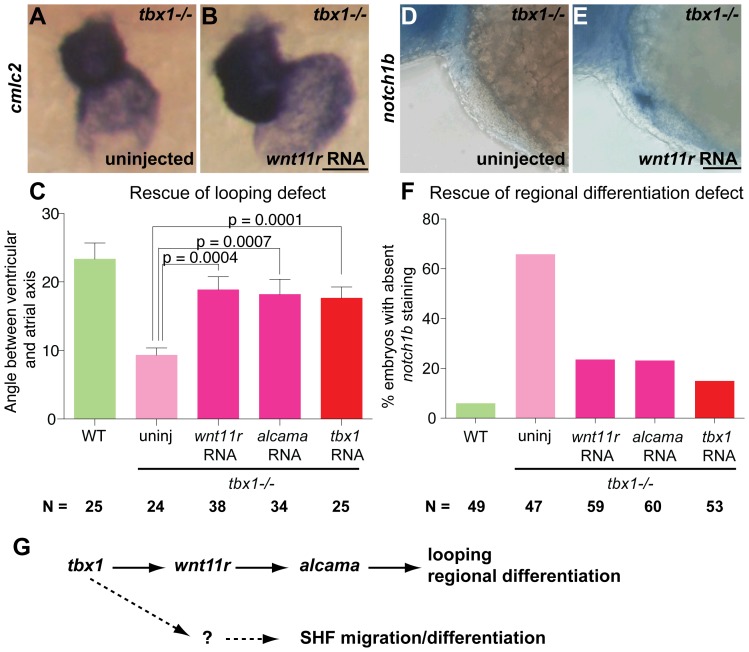
*tbx1* functions through *wnt11r* to regulate heart looping and regional differentiation. ISH for *cmlc2* (A, B) and *notch1b* (D, E) showing rescue of looping (B) and regional differentiation defect (E) at 48 hpf after injection of 26 pg *wnt11r* RNA at the 1-cell stage. Plots in C and F quantify the rescue of looping and regional differentiation defect after the indicated injections. WT siblings are represented in green and uninjected *tbx1^−/−^* mutants in pink, with injected mutants in dark pink or red. Scale bars: 50 µm. N indicates the total number of embryos from 3 experiments. (G) Outline of the working model, where *tbx1* regulates *wnt11r*; *wnt11r* regulates *alcama*, which in turn regulates heart looping and regional differentiation. *tbx1* regulates migration/differentiation of SHF cells by an independent mechanism.

We further tested whether *wnt11r* and *alcama* function downstream of *tbx1* by rescue experiments. If cardiac defects in *tbx1^−/−^* mutants are caused specifically by suppression of the *wnt11r-alcama* pathway, ectopic expression of *wnt11r* or *alcama* should rescue cardiac defects in *tbx1^−/−^* mutants. We injected *wnt11r* or *alcama* RNA into 1-cell stage *tbx1^−/−^* mutants at concentrations that do not produce a phenotype in WT embryos (see methods). Injected mutants were assessed for heart looping (*cmlc2* ISH, Fig. 8B) at 52 hpf. *tbx1* RNA was injected as a positive control for rescue. Injection of either *wnt11r* RNA or *alcama* RNA increased the angle between the ventricular and atrial axes to nearly WT levels (18° in RNA injected *tbx1^−/−^* mutants versus 9° in uninjected *tbx1^−/−^* mutants and 23° in WT) (Fig. 8C). This data demonstrates that *wnt11r* and *alcam* do indeed function downstream of *tbx1* to regulate heart looping in zebrafish. Similarly, mutants were assessed for regional differentiation defects (*notch1b* ISH, Fig. 8E) following injection of *wnt11r* or *alcama* RNA. Injection of either RNA decreased the percentage of larvae with absent *notch1b* staining (Fig. 8F). Partial rescue of *tbx1^−/−^* mutants by RNA injection substantiates our model whereby *tbx1*, *wnt11r* and *alcama* function in a linear pathway regulating zebrafish cardiac development. Partial rescue can be explained by differences in dosage and timing of expression, degradation of RNA by late stages, or other downstream targets of *tbx1* that remain unexplored to date. These data taken together with our expression analysis support our hypothesis whereby *tbx1* activates *wnt11r*, which then activates *alcam*a to regulate heart looping and differentiation (Fig. 8G).

## Discussion

### *tbx1* is required for heart formation, cardiomyocyte shape and differentiation

*Tbx1* has been identified as the gene critical for regulating DGS etiology However, the molecular and cellular defects caused by loss of *tbx1* have not been well characterized. Using the zebrafish *tbx1^−/−^* mutant, we have demonstrated that similar to mice, zebrafish *tbx1* is required for normal heart development. Zebrafish *tbx1^−/−^* mutants have improperly looped hearts, undifferentiated BA and other morphological defects. These defects may be a milder presentation of the conotruncal defects and ventricular septal defects in DGS patients and mouse *Tbx1−/−* mutants. Indeed, genes regulating zebrafish heart looping such as *Bmp4*, *Tbx2*, *Tbx3*, *Tbx5*, *Tbx20*, and *Wnt11r*, are associated with conotruncal defects in humans or mice, but not looping defects [58], [59], [60], [61], [62].

Furthermore, data indicates that OFT defects result from a failure of OFT myocardial wall to rotate [63], suggesting that myocardial wall rotation might be the final stage of the looping process that is affected in mouse mutants and DGS patients. This would explain the intersection of genes involved in the two processes. Hence, improper looping and BA morphology in zebrafish may be a milder presentation of the conotruncal defects in DGS patients. Our data reveals a new role for *tbx1* in regulating heart looping thereby contributing to the conotruncal and septal development of the heart. This may provide a new mechanism by which loss of Tbx1 contributes to conotruncal cardiac defects in DGS.

The looping defect, taken together with the weaker ventricular contractions and slower heart rate in *tbx1^−/−^* mutants, suggests that *tbx1* is required for proper heart formation. Despite the defects in heart formation, the early stages of cardiogenesis proceed normally in *tbx1* mutants. Atrial and ventricular fates are assigned normally in the *tbx1^−/−^* mutant as indicated by normal *cmcl2*, *vmhc* and *amhc* ISH staining (Fig. 1, S3), and normal antibody staining for S46 and MF20 (data not shown). Given the expression of *tbx1* in cardiac mesodermal progenitors from the fusion stage (Fig. S1A), this finding was surprising. However, it is consistent with mouse data [12] and suggests that *tbx1* is not required for heart specification and early development.

While cells are specified properly our data show that cell shape is disrupted in *tbx1* mutants. In *tbx1^−/−^* mutants cells fail to change from isotropic to anisotropic shape in the outer curvature. Other zebrafish mutants/morphants with looping defects share the observed defects in cell shape [46], [50], [52]. This cell shape defect combined with reduction in cell number may contribute to looping defects by changing cell-cell interaction. Indeed, cross-sections of *tbx1^−/−^* mutant hearts reveal an increase in extracellular spaces compared to WT siblings. This observation is important, as increased extracellular spaces have been observed in *wnt11r* and *alcama* morphants in *Xenopus* [15], [16]. Decreased cell adhesion due to down-regulation of the adhesion molecule *alcama*, may be causing the increase in extracellular spaces in *tbx1^−/−^* mutants. Alternatively, the increased extracellular spaces may lead to altered cell polarity or may preclude changes in cell shape or differentiation. These data indicate that *tbx1* is required for induction of morphological, proliferative and shape changes in cardiomyocytes.

An additional factor contributing to the heart defects observed in *tbx1^−/−^* is a loss of tissue differentiation. Cardiomyocytes fail to differentiate into the OFT and AVC as indicated by down regulation of *bmp4*, *versican* and *notch1b*. In addition mis-expression of *anf* also corroborates this conclusion. Absent expression of *bmp4*, *versican* and *notch1b* in *tbx1^−/−^* mutants is in striking contrast to other looping mutants, where these markers fail to be restricted to their respective domains [46], [49], [50], [52]. Hence we propose that *tbx1* regulation of heart looping is distinct from genes such as *tbx2a*, *tbx3b* and *nkx2.5*, which also regulate this process. An unresolved question is whether differentiation of the heart regions contributes to looping or vice versa.

### *tbx1* regulates differentiation of the SHF and BA

DGS patients and *Tbx1−/−* mice have OFT defects such as persistent truncus arteriosus [2]. Moreover, *Tbx1* is expressed in the mouse SHF and regulates SHF contribution to the OFT [12]. The zebrafish SHF has been recently described [31], [32] but *tbx1* expression in SHF cells has not been described so far. However, similarly to data in mouse, zebrafish *tbx1^−/−^* mutants have a smaller BA, indicating decreased contribution of SHF to the arterial pole of the heart. In addition, absent *eln2* expression and DAF-2DA staining indicates that smooth muscle in the BA is undifferentiated. In addition to the BA, the ventricle in *tbx1^−/−^* mutants is also small and dysmorphic, with a severe reduction in total cardiomyocyte number. While our investigation of the BA defect in *tbx1^−/−^* mutants was ongoing, absence of *tbx1* was reported to reduce differentiation and incorporation of SHF-derived cells to the arterial pole of the heart [32]. These results are complementary to our data, indicating that *tbx1* is required for SHF cell incorporation into the BA and subsequent differentiation of smooth muscle cells.

Reduced incorporation of cardiomyocytes from the SHF in zebrafish *tbx1^−/−^* mutant hearts suggests that the processes involved in heart development are conserved through evolution. Furthermore, the BA defects in zebrafish are consistent with conotruncal and septal defects observed in DGS patients and *Tbx1−/−* mice. However, as opposed to humans and mice, zebrafish do not develop ventricular hypertrophy as a compensatory mechanism to outflow tract defects. The defects in looping, differentiation, and heart rate emerge at 48 hpf, **before** the first detectable time point for outflow tract defect (72 hpf), suggesting that the ventricular defect is not secondary to the outflow tract defect.

While *Tbx1*'s role in regulating proliferation and contribution of SHF cells has been studied [12], our data reveals a role for *tbx1* in regulating heart looping. We postulate that the conotruncal and septal defects in DGS may be a more severe manifestation of the defects in SHF cell incorporation and the defects in morphological movements of looping during development.

### *wnt11r* functions downstream of *tbx1*

Although *Wnt11* signaling has been shown to be important for specification of cardiac fate and *Wnt11−/−* mutants have OFT defects [19], [20], [25], *Tbx1* and *Wnt11* signaling pathways have not been linked to date. Unlike mouse and chicken embryos, *Xenopus* and zebrafish embryos do not express *wnt11* in the developing heart [15]. *wnt11r*, a second *wnt11* gene in *Xenopus* and zebrafish, with high homology to human and chicken *Wnt11*, mediates non-canonical *Wnt* signaling and is necessary for normal heart morphogenesis. In *Xenopus*, *wnt11r* starts to be expressed just prior to fusion of cardiac progenitors and subsequently continues to be expressed in the heart tissue. It was later discovered that *alcama* regulates cardiac looping and functions downstream of *wnt11r* in *Xenopus* [16].

The role of *wnt11r* in zebrafish heart morphogenesis has not been previously characterized. We show that similarly to *Xenopus*, zebrafish *wnt11r* is first expressed at 21somites just prior to cardiac progenitor fusion and continues to be expressed as the heart develops. Our data shows that *wnt11r^−/−^* mutants have similar looping and regional differentiation defects as observed in *tbx1^−/−^* mutants, and that *wnt11r* is down-regulated in *tbx1^−/−^* mutant hearts. Furthermore, our non-allelic non-complementation assay indicates that *tbx1* and *wnt11r* function in the same pathway. Knockdown of *alcama*, a gene demonstrated to be downstream of *wnt11r*, also presents with looping and regional differentiation defects. Importantly, we were able to rescue the looping and differentiation defects in *tbx1^−/−^* mutants by injection of *wnt11r* and/or *alcama* RNA. All these data lead us to our working model that *tbx1* regulates *wnt11r*, *wnt11r* regulates *alcama*, which in turn regulates heart looping and differentiation (Fig. 8G). Elucidation of new players in the pathway may help in developing future therapies for patients with DGS and other cardiac defects.

## Conclusions

In summary, this study demonstrates that zebrafish *tbx1^−/−^* mutants have defects in heart looping and function. This is the first demonstration that *tbx1* regulates differentiation and shape of cardiac cells derived from PHF. In addition, we describe *tbx1*'s role in regulating total cardiomyocyte number via PHF cell proliferation that may be compounded by lack of contribution of SHF cells in *tbx1^−/−^* mutants. Importantly, we have identified *wnt11r* and *alcama* as novel mediators of the *tbx1* pathway. We show for the first time that in zebrafish *wnt11r* ^−/−^ mutants and *alcama* morphants have heart looping and differentiation defects similar to *tbx1* ^−/−^ mutants, and our expression and non-complementation assay confirms that *wnt11r* and *tbx1* function in the same pathway. Moreover, these defects can be rescued by over-expression of *wnt11r* and/or *alcama* in *tbx1^−/−^* mutants, suggesting that they function downstream of *tbx1*. Our data support a model whereby *tbx1* regulates heart looping and differentiation via *wnt11r* and *alcama*. These findings are an important contribution to our understanding of *tbx1* signaling and heart development.

## Supporting Information

Figure S1 ***tbx1*** **expression and heart shape defect of** ***tbx1^−/−^*** **1arvae.** ISH for *tbx1* in WT embryos showing expression in the fusing heart fields (21 somites, A), linear heart tube (24 hpf, B) and the looped heart (48 hpf, C). A and B are dorsal views and C is a head-on view. Arrowheads in A and B point towards pharyngeal pouches and the cardiac cells are outlined in A–C. (D) Schematic of head-on view of 48 hpf heart. Arrow indicates the AVC; a, atrium; v, ventricle. Dotted red lines indicate the widths at the ends and at the widest part of the ventricle and atrium. The longitudinal axes (shown in green) were drawn by joining the midpoints m and n in ventricle and midpoints p and q in atrium. mo and pr were measured for length of ventricle and atrium, respectively (see Materials for details). (E) Measurement of lengths of the ventricle and atrium in WT and *tbx1^−/−^* mutants. (F, G) Measurement of the widths of the ventricles at 48 hpf and atriums at 72 hpf in *tbx1^−/−^* mutants and WT siblings in the expanded and contracted states. WT siblings are represented in green and *tbx1^−/−^* mutants in pink. N indicates the total number of embryos represented in the plot. Scale bars: 50 µm.
(TIF)

Figure S2 **Proliferation defects in** ***tbx1^−/−^*** **mutants.** (A, B) Confocal projections of hearts from *cmlc2:gfp* (all heart cells are green) embryos at 33 hpf stained with phospho-histone 3 antibody (proliferating cells are red). The insets in A and B show a magnified view of a co-labeled cell (yellow). (C, D) Plots showing the total (C) and proliferating (D) number of cells in WT and *tbx1^−/−^* mutants. N is the total number of embryos. Arrows point to the AVC; v, ventricle; a, atrium. Scale bar: 25 µm.
(TIF)

Figure S3 **Chambers are specified correctly in** ***tbx1^−/−^*** **mutants.** ISH analysis at 48 hpf for *vmhc* (A, C) and *amhc* (B, D) shows that the chambers are correctly specified in *tbx1^−/−^*mutants. The dotted lines indicate the heart boundary. Scale bars: 50 µm.
(TIF)

Figure S4 ***tbx1^−/−^*** **embryos have defects in heart performance.** (A) Measurement of ventricular contractility as shortening fraction [(width at diastole – width at systole)/width at diastole] shows no difference between WT and *tbx1^−/−^* embryos. (B) Determination of atrium contractility between WT and *tbx1^−/−^* embryos. The stroke volume (ventricular volume [diastole – systole]) plotted in C is unaffected in *tbx1^−/−^* embryos, but the heart rate is decreased, shown in D. N = 19 in all experiments. WT siblings are represented in green and *tbx1^−/−^* mutants in pink. p-values are reported under each measurement.
(TIF)

Figure S5 **Candidate gene analysis for** ***tbx1^−/−^*** **mutants.** Whole mount lateral views (A, B, F, G) and head-on views (C–E, H–J) of 48 hpf embryos stained for known genes that affect heart looping. Expression of *tbx2a* (A, F), *tbx3b* (B, G), *tbx5* (C, H) and *tbx20* (D, I) was unaffected between WT siblings and *tbx1^−/−^* mutants. (E, J) Dissected hearts from 48 hpf embryos show down-regulated expression of *wnt11r* in the mutant (J) versus WT (E). The heart boundary is shown by the red dotted line. Arrows point to the AVC. Scale bars: 50 µm.
(TIF)

Figure S6 **Unaffected BA in** ***wnt11r ^−/−^*** **mutants.** (A, B) Lateral and ventral views of 72 hpf larva showing normal *eln2* and DAF-2DA staining respectively in *wnt11r^−/−^* mutants. Scale bars: 50 µm.
(TIF)
