# Supplementary figures and images for: DiGeorge Syndrome Gene tbx1 Functions through wnt11r to Regulate Heart Looping and Differentiation
Source: PLoS One. 2013 Mar 22;8(3):e58145. doi: 10.1371/journal.pone.0058145 (PMC3606275; doi:10.1371/journal.pone.0058145)

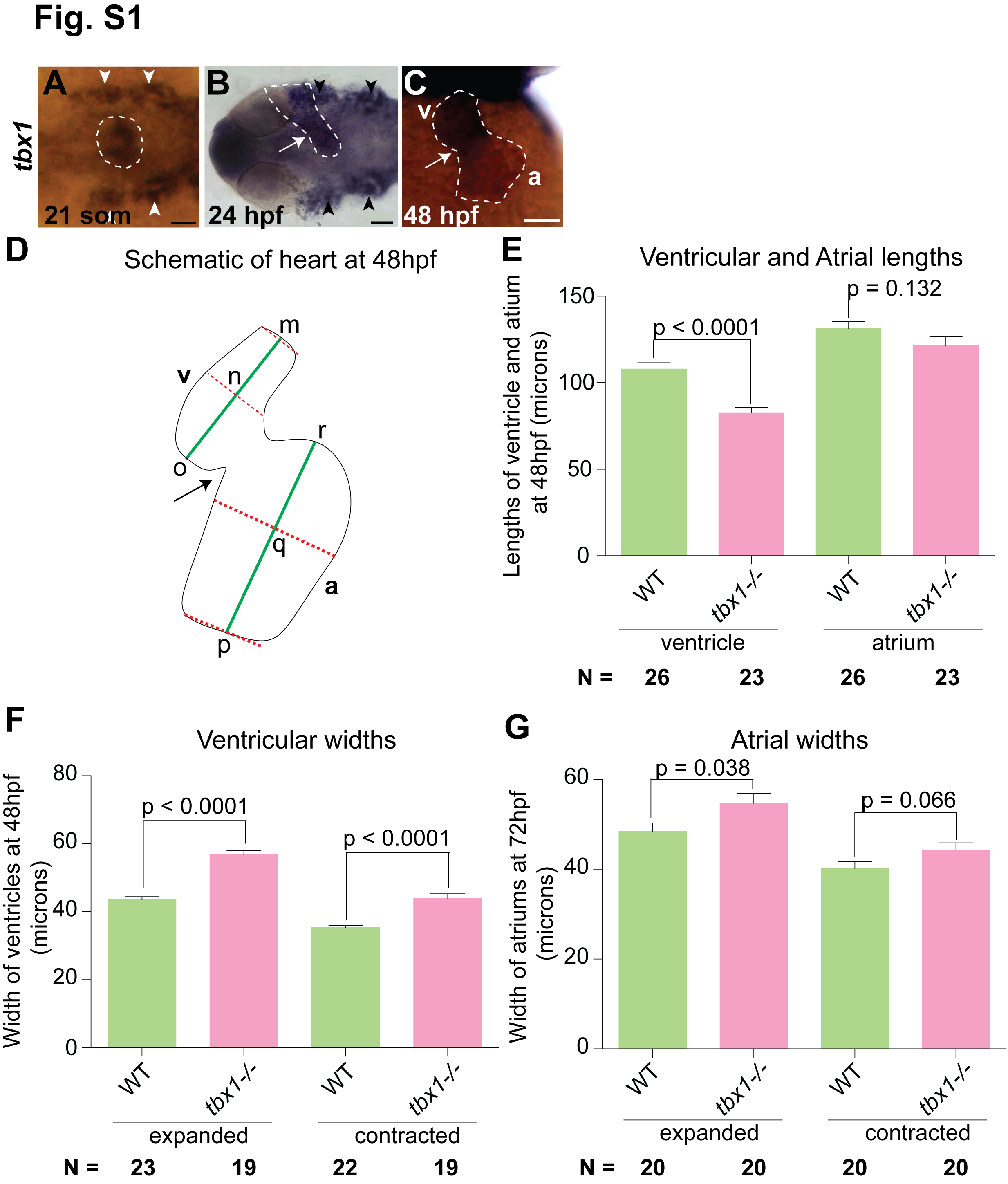

Supplement: Figure S1 — tbx1 expression and heart shape defect of tbx1−/− 1arvae. ISH for tbx1 in WT embryos showing expression in the fusing heart fields (21 somites, A), linear heart tube (24 hpf, B) and the looped heart (48 hpf, C). A and B are dorsal views and C is a head-on view. Arrowheads in A and B point towards pharyngeal pouches and the cardiac cells are outlined in A–C. (D) Schematic of head-on view of 48 hpf heart. Arrow indicates the AVC; a, atrium; v, ventricle. Dotted red lines indicate the widths at the ends and at the widest part of the ventricle and atrium. The longitudinal axes (shown in green) were drawn by joining the midpoints m and n in ventricle and midpoints p and q in atrium. mo and pr were measured for length of ventricle and atrium, respectively (see Materials for details). (E) Measurement of lengths of the ventricle and atrium in WT and tbx1−/− mutants. (F, G) Measurement of the widths of the ventricles at 48 hpf and atriums at 72 hpf in tbx1−/− mutants and WT siblings in the expanded and contracted states. WT siblings are represented in green and tbx1−/− mutants in pink. N indicates the total number of embryos represented in the plot. Scale bars: 50 µm. (TIF) [file pone.0058145.s001.tif]

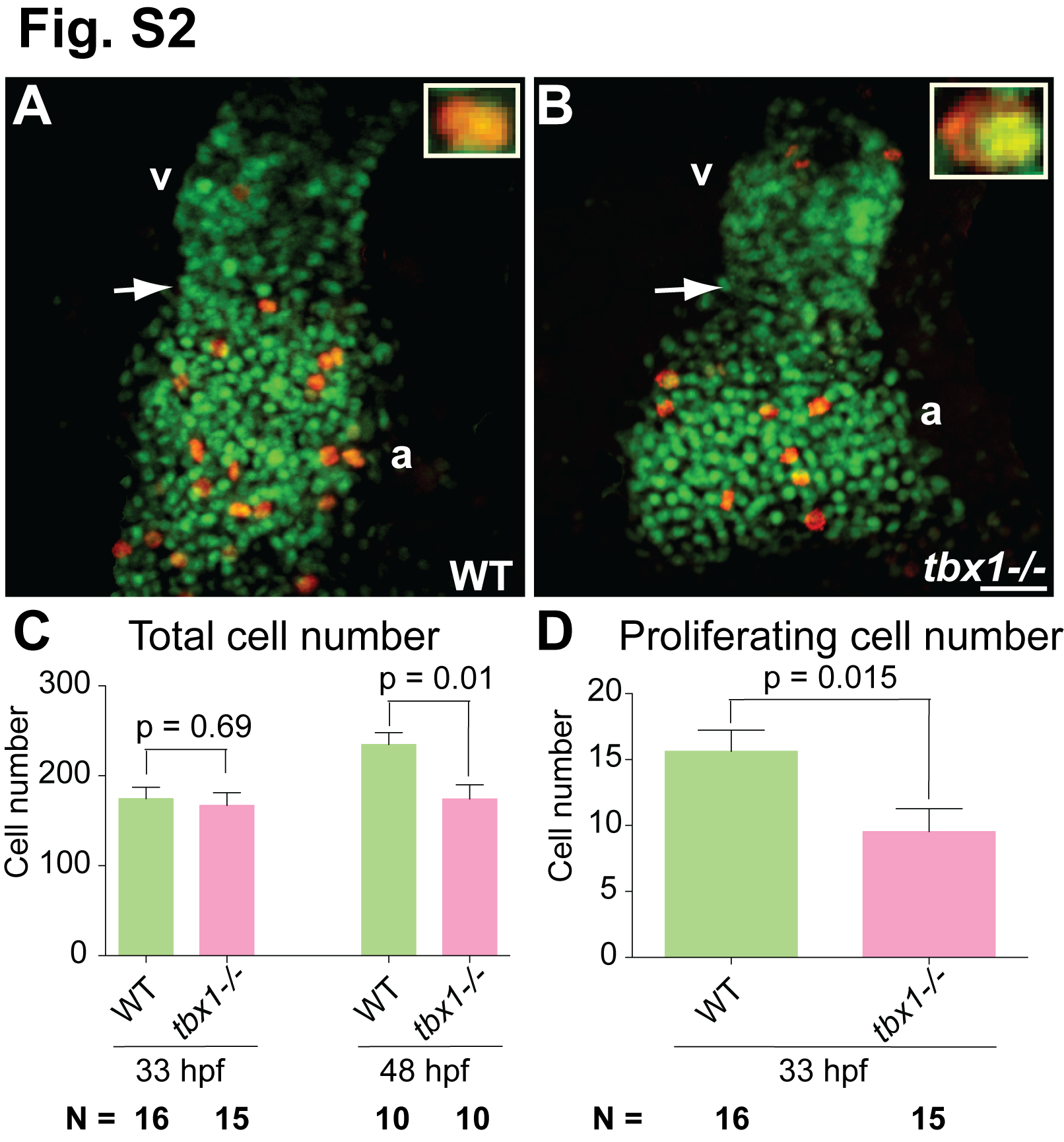

Supplement: Figure S2 — Proliferation defects in tbx1−/− mutants. (A, B) Confocal projections of hearts from cmlc2:gfp (all heart cells are green) embryos at 33 hpf stained with phospho-histone 3 antibody (proliferating cells are red). The insets in A and B show a magnified view of a co-labeled cell (yellow). (C, D) Plots showing the total (C) and proliferating (D) number of cells in WT and tbx1−/− mutants. N is the total number of embryos. Arrows point to the AVC; v, ventricle; a, atrium. Scale bar: 25 µm. (TIF) [file pone.0058145.s002.tif]

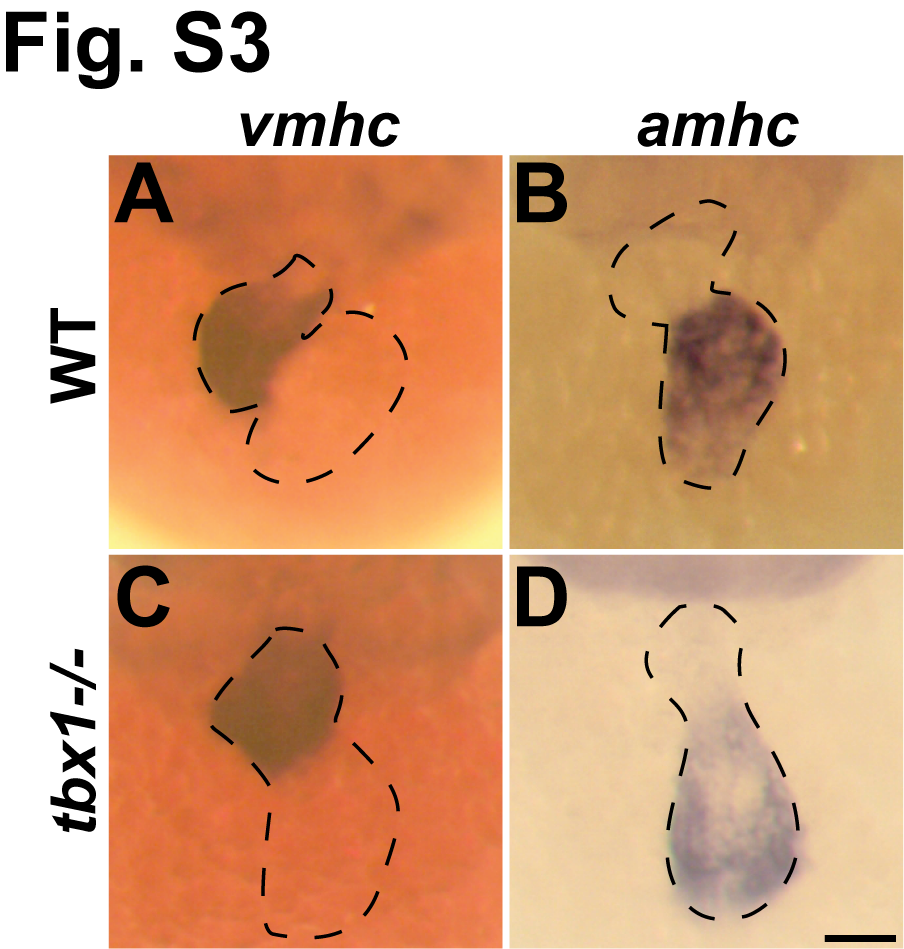

Supplement: Figure S3 — Chambers are specified correctly in tbx1−/− mutants. ISH analysis at 48 hpf for vmhc (A, C) and amhc (B, D) shows that the chambers are correctly specified in tbx1−/−mutants. The dotted lines indicate the heart boundary. Scale bars: 50 µm. (TIF) [file pone.0058145.s003.tif]

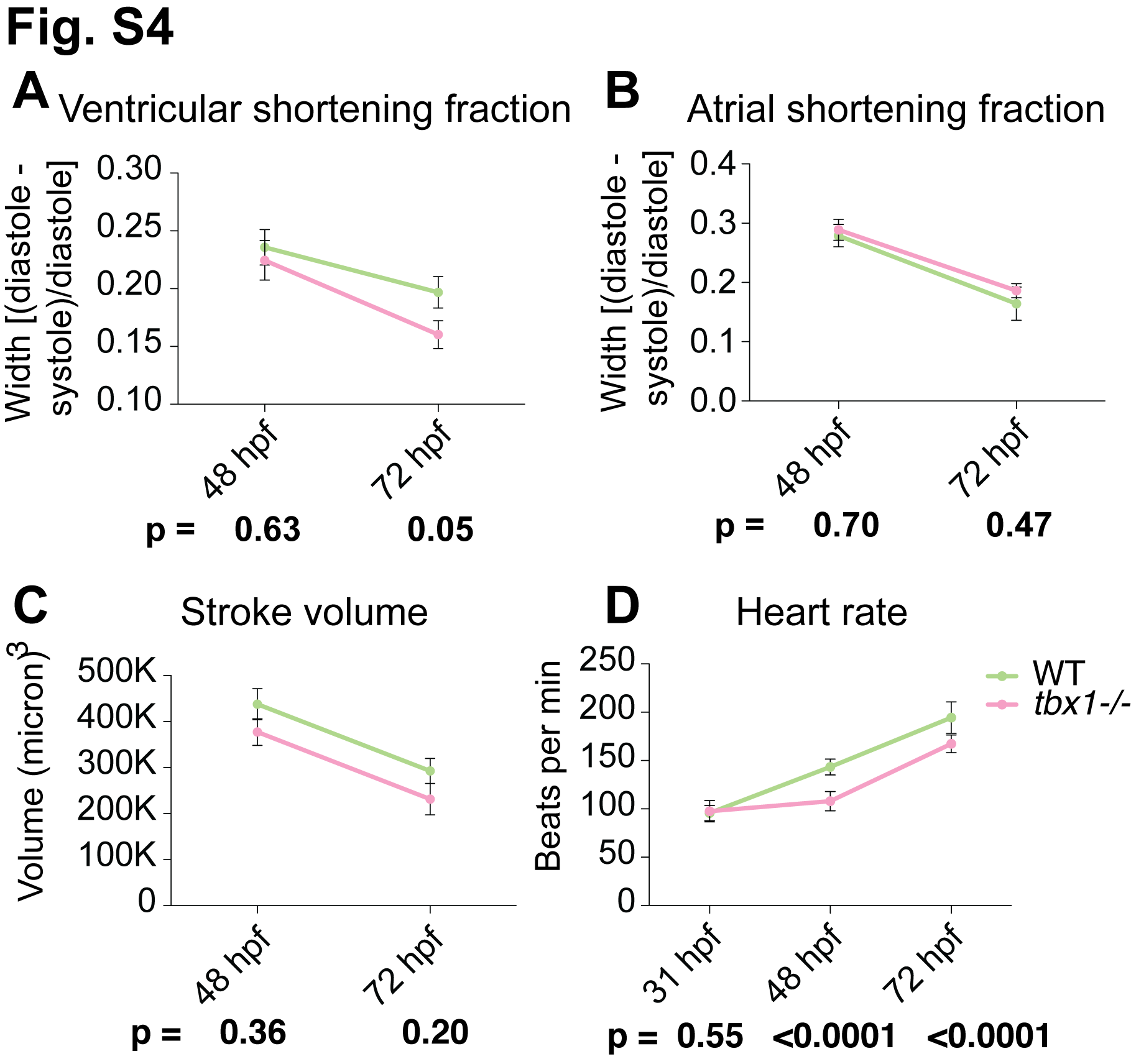

Supplement: Figure S4 — tbx1−/− embryos have defects in heart performance. (A) Measurement of ventricular contractility as shortening fraction [(width at diastole – width at systole)/width at diastole] shows no difference between WT and tbx1−/− embryos. (B) Determination of atrium contractility between WT and tbx1−/− embryos. The stroke volume (ventricular volume [diastole – systole]) plotted in C is unaffected in tbx1−/− embryos, but the heart rate is decreased, shown in D. N = 19 in all experiments. WT siblings are represented in green and tbx1−/− mutants in pink. p-values are reported under each measurement. (TIF) [file pone.0058145.s004.tif]

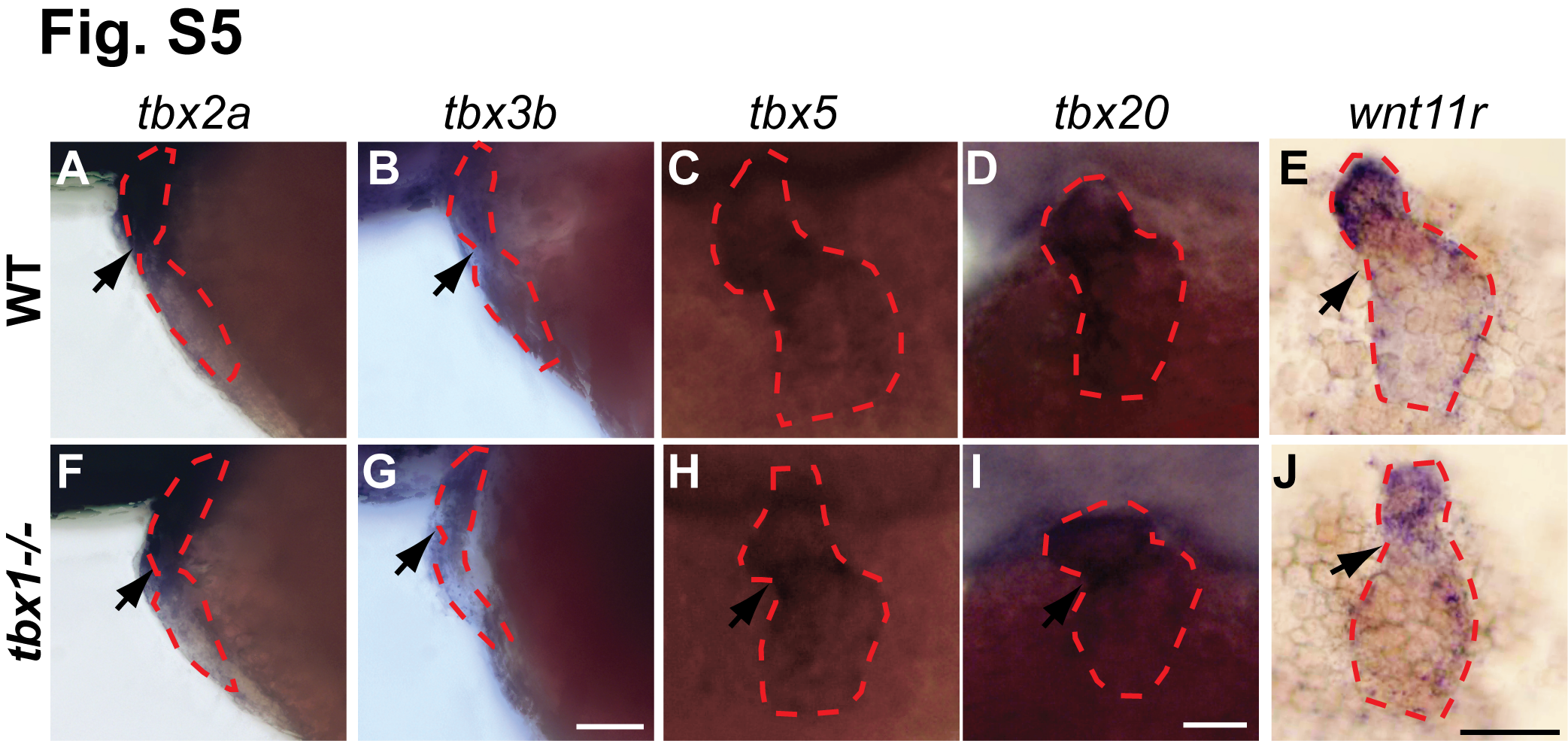

Supplement: Figure S5 — Candidate gene analysis for tbx1−/− mutants. Whole mount lateral views (A, B, F, G) and head-on views (C–E, H–J) of 48 hpf embryos stained for known genes that affect heart looping. Expression of tbx2a (A, F), tbx3b (B, G), tbx5 (C, H) and tbx20 (D, I) was unaffected between WT siblings and tbx1−/− mutants. (E, J) Dissected hearts from 48 hpf embryos show down-regulated expression of wnt11r in the mutant (J) versus WT (E). The heart boundary is shown by the red dotted line. Arrows point to the AVC. Scale bars: 50 µm. (TIF) [file pone.0058145.s005.tif]

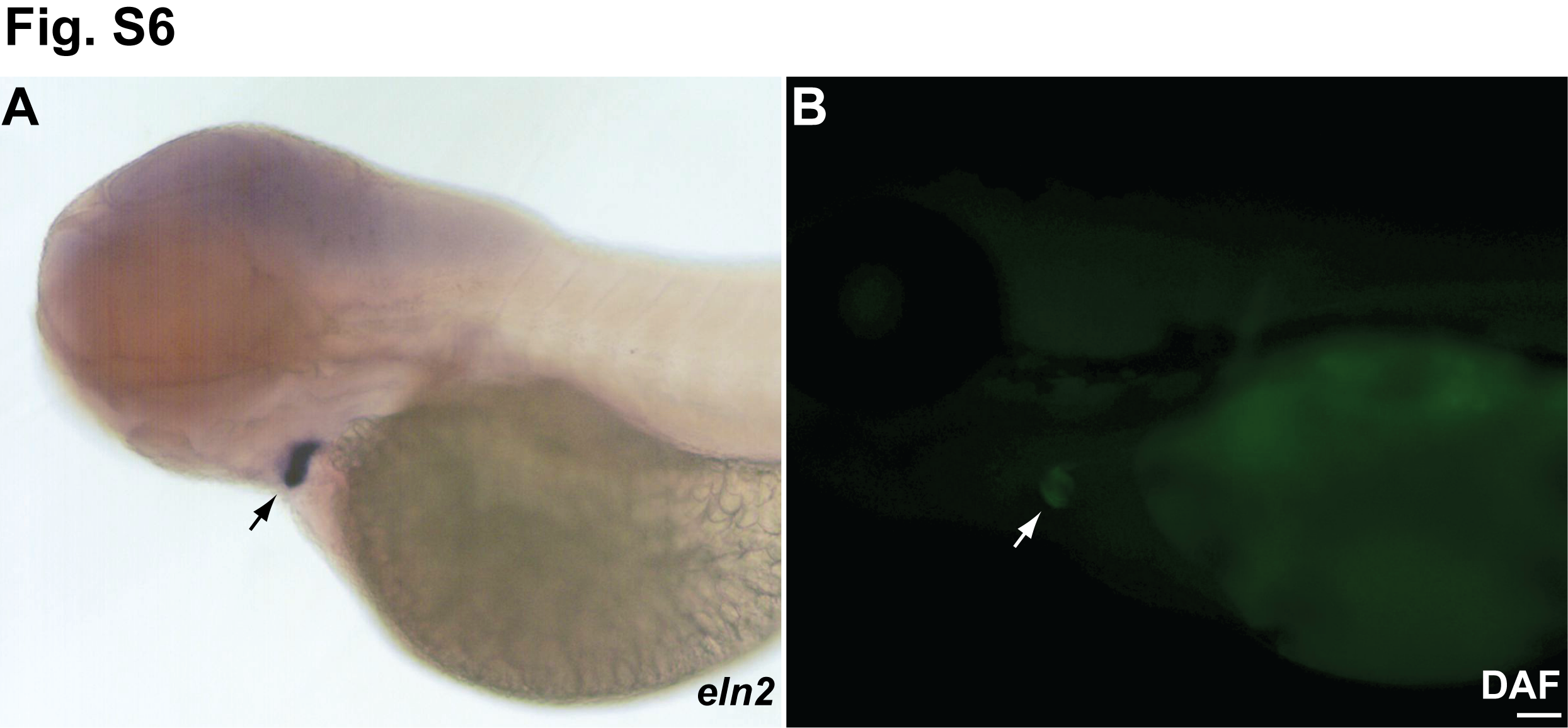

Supplement: Figure S6 — Unaffected BA in wnt11r −/− mutants. (A, B) Lateral and ventral views of 72 hpf larva showing normal eln2 and DAF-2DA staining respectively in wnt11r−/− mutants. Scale bars: 50 µm. (TIF) [file pone.0058145.s006.tif]
